# Supplementary material for: Plasma electron acceleration driven by a long-wave-infrared laser
Source: Nat Commun. 2024 May 13;15:4037. doi: 10.1038/s41467-024-48413-y (PMC11519596; doi:10.1038/s41467-024-48413-y)
Supplement: Supplementary file 1 — Supplementary Information [file 41467_2024_48413_MOESM1_ESM.pdf]

Supplementary Information

Plasma electron acceleration driven by a long-wave-infrared laser

R. Zgadzaj,[1] J. Welch,[1] Y. Cao,[1] L. D. Amorin,[2] A. Cheng,[2] A. Gaikwad,[2] P. Iapozzutto,[2] P. Kumar,[2] V. N. Litvinenko,[2] I. Petrushina,[2] R. Samulyak,[2] N. Vafaei-Najafabadi,[2] C. Joshi,[3] C. Zhang,[3] M. Babzien,[4] M. Fedurin,[4] R. Kupfer,[4] K. Kusche,[4] M. A. Palmer,[4] I. V. Pogorelsky,[4] M. N. Polyanskiy,[4] C. Swinson,[4] and M. C. Downer[1]

[1]*University of Texas at Austin, 1 University Station C1600, Austin, Texas 78712 USA*
[2]*Stony Brook University, Stony Brook, New York 11794 USA*
[3]*University of California at Los Angeles, Los Angeles, California 90024 USA*
[4]*Brookhaven National Laboratory, Upton, New York 11973 USA*

Simulated wakes with stationary ions. In the section entitled "Results/Generation of self-modulated wakes" of the main text we stated that simulations showed a strong influence of including ion motion on the structure and evolution of the self-modulated wakes compared to those produced in a plasma with stationary ions. Consequently all simulation results shown in the main text used moving ions. In Supplementary Fig. 1, for comparison, we show one example of a self-modulated wake structure simulated using the stationary ion approximation. The excitation conditions and the instant of time (stated in the caption) are identical to those used for the moving-ion simulation shown in Fig. 1d of the main text. Yet the wake structure is very different. Most prominently, a deep ion channel forms in Fig. 1d that is completely absent in Supplementary Fig. 1. Moreover in Fig. 1d the wake is stronger and the drive laser pulse more deeply modulated. See Ref. [1] for additional comparisons of stationary vs. moving ion simulations.

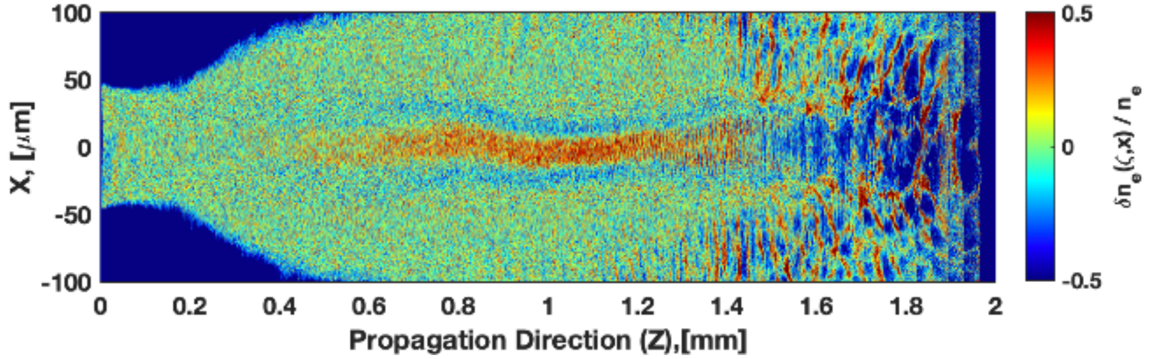

Supplementary Fig. 1. Simulated self-modulated wake structure with stationary hydrogen ions. 2D wake profile $\delta n_e(x,z)/n_e$ at the instant that 4 J, 2 TW pump pulse vacuum-focused at z = 0.1 mm has propagated to z = 1.7 mm in a hydrogen gas jet of plateau density $n_e = 5 \times 10^{17}$ cm^{-3}. This result can be compared and contrasted with Fig. 1(d) of the main text, which depicts the wake formed with identical excitation conditions, but with moving ions.

Supplementary electron spectrometer data. Figure 7 of the main manuscript presents electron spectrometer data for five shots. They illustrate two examples that yielded exponential electron energy distributions (rows 1-2) and three with peaked, quasimonoenergetic features (rows 3-5). Supplementary Fig. 2 presents additional raw data showing examples of both types of spectra.

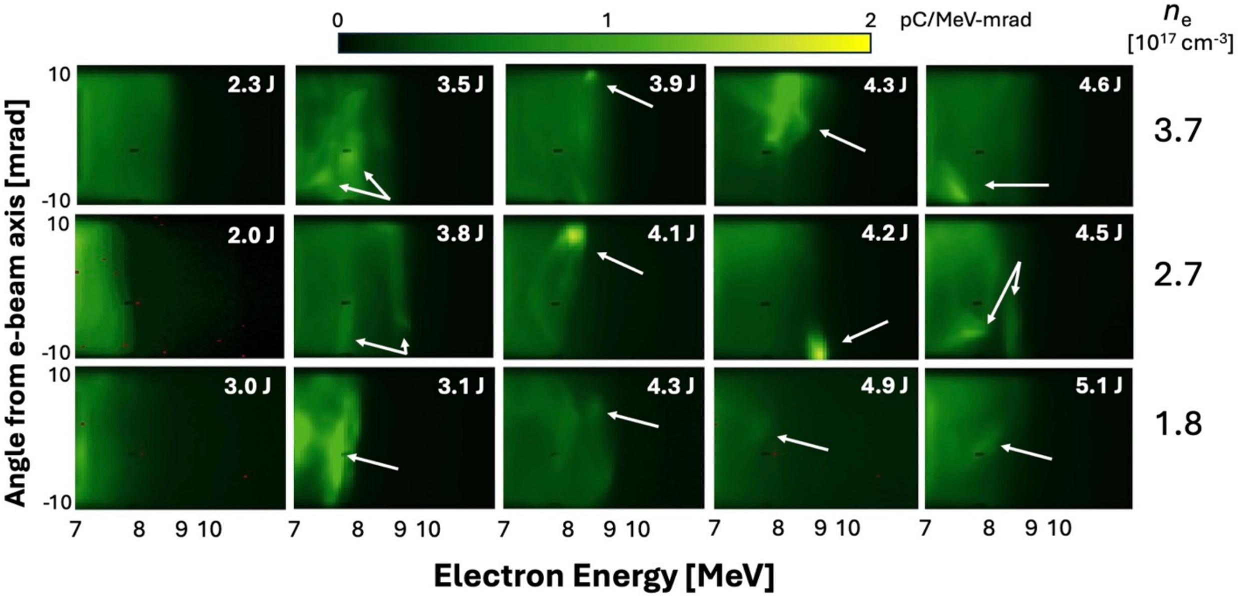

Supplementary Fig. 2. Electron energy and angular distributions. Additional examples of LANEX luminescence images from magnetic spectrometer configured to record electrons with energies $7 < E_e < 15$ MeV, from CO_2-laser-driven WFAs in plasma of density 3.7 (row 1), 2.7 (row 2), and 1.8 (row 3) × 10^{17} cm^{-3}. Spectra in column 1 show exponentially decreasing (e^{-E_e/k_BT_e}) yield with negligible angular variation within the range detected. Spectra in columns 2-5 include collimated quasi-monoenergetic peaks, which are highlighted by white arrows. Numbers in upper right corner of each panel indicate the measured energy of the 2-ps CO_2 laser pulse.

Vacuum Focused Spatial Profiles of the Terawatt CO_2 Laser. In section Methods/CO_2 Laser of the main text, we refer to Supplementary Information on focus profiles, spectra, and durations of terawatt CO_2 laser pulses. Supplementary Figure 3a,b shows false-color images of the vacuum-focused intensity profiles of seven 2 ps pulses as their energy $\mathcal{E}_L$ varied from ~ 0.5 J (left) to ~ 5 J (right), plotted on a) linear and b) logarithmic intensity scales relative to each pulse's peak intensity I_L. To obtain the images, a BaF_2 lens imaged the vacuum-focused CO_2 beam to a pyroelectric camera (Ophir Spiricon Pyrocam IV) with ~ 50× magnification, after reflections from several glass or NaCl windows reduced the intensity by a calibrated amount to the camera's linear response range. Supplementary Fig. 3c plots I_L (obtained from the scaled camera response and measured $\mathcal{E}_L$, duration τ_L, and spot size w_0) vs. $\mathcal{E}_L$ for these 7 (plus 12 additional) shots over the same $\mathcal{E}_L$ range. For $\mathcal{E}_L < 3$ J, no change in the profiles was observed, even in the wings, as energy varied from shot-to-shot (Supplementary Fig. 3b). Moreover I_L varied linearly with $\mathcal{E}_L$ (Supplementary Fig. 3c), further corroborating focal stability. For $\mathcal{E}_L > 3$ J, slight variations became evident in the wings (Supplementary Fig. 3b), and intensity deviated from a linear increase with energy (Supplementary Fig. 3c). This sub-linear I_L ($\mathcal{E}_L$) indicates a slight degradation in the focused profile, attributable to the aforementioned nonlinear optical interactions in the air-filled beam transport line. As an example, the inset of Supplementary Fig. 3c compares a Gaussian profile of $w_0 = 27.5$ µm, which fits with < 5% error to the average focused radial profiles of sub-3 J pulses, to the profile of the 4 J pulse in (a). The latter is broadened by ~ 15%, consistent with an intensity ~ 25% below the linear trend.

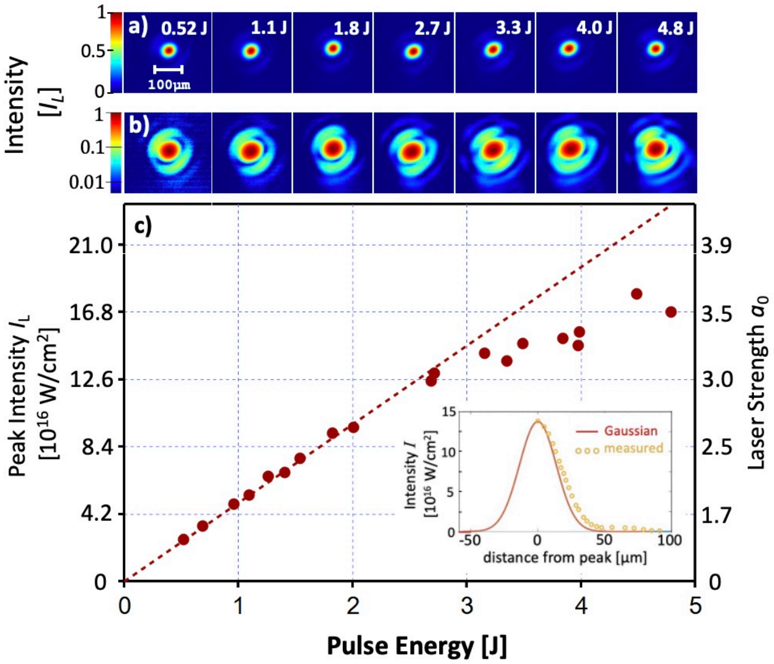

Supplementary Fig. 3. Focus profiles of 2 ps CO_2 laser pulses. **(a,b)** Images of vacuum-focused intensity profiles of seven $0.5 \lesssim \mathcal{E}_L \lesssim 5$ J pulses, plotted on (a) linear and (b) logarithmic color scales relative to each pulse's peak intensity I_L. **c)** Plot of I_L vs. $\mathcal{E}_L$ for 19 pulses. I_L of the lowest $\mathcal{E}_L$ pulse was evaluated from $I_L = 2\mathcal{E}_L/\pi w_0^2 \tau_L$ using directly measured values $\mathcal{E}_L = 0.52$ J and $\tau_L = 2$ ps, and $w_0 = 27.5$ μm from a Gaussian fit to the measured intensity profile. For higher $\mathcal{E}_L$, measured $\tau_L = 2$ ps remained constant, and peak fluence $I_L = 2\mathcal{E}_L/\pi w_0^2$ was scaled in proportion to camera counts within a small circle enclosing the peak of each pulse to determine I_L. Inset: Comparison of average measured radial profile of 4 J pulse profile from (a) (open circles) with Gaussian profile of $w_0 = 27.5$ μm (dark red curve) used in simulations.

Spectra of CO_2 Laser Pulses. Initial wake generation experiments used a 0.5 TW configuration before the latest upgrade of the CO_2 laser. The main difference of the old system compared to the present configuration was the use of regular (not isotopically enriched) CO_2 in the final amplifier. The strongest laser transition of a regular CO_2 amplifier (10R rotational-vibrational branch) is centered at 10.3 μm and is strongly modulated by rotational bands (see Supplementary Fig. 4). This spectrum is narrower and weaker than the 9R branch of a mixed-isotope amplifier at 9.2 μm (see Fig. 4 of Ref. [2]) resulting in a longer ($\tau_L \approx 4$ ps) pulse. The broader, more uniform spectrum of the 9R branch amplifier also reduces spectral gain narrowing compared to the 10R configuration. The spectrum of the amplified pulse, and correspondingly, its temporal structure, are defined by the gain spectrum. As such, they do not exhibit pulse-to-pulse or day-to-day variations that are substantial for the experiment. Focused profiles in the 0.5 TW, 4 ps configuration closely resembled those of $\tau_L \approx 2$ ps, $\mathcal{E}_L < 3$ J pulses shown in Supplementary Fig. 3(a,b), and were also very stable from shot-to-shot.

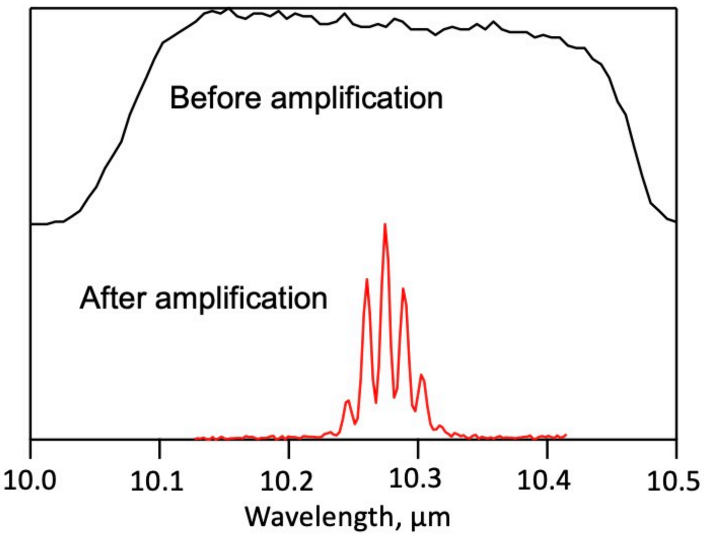

Supplementary Fig. 4. **Spectra of CO_2 laser pulses**. Spectrum of compressed 4 ps, 10.3 μm pulses, showing strong rotational modulation and gain narrowing. This should be compared and contrasted with spectra of compressed 2 ps, 9.2 μm pulses, shown in Fig. 4 of Ref. [2] with weak and strong gain saturation.

Duration of CO_2 laser pulses. The temporal profile of the LWIR laser pulse was measured using a single-shot intensity autocorrelation technique [2]. Fig. 6 of Ref. [2] shows typical autocorrelations traces and temporal pulse profiles in the 2 ps, 2 TW configuration, for which pulse duration τ_L varied insignificantly from shot-to-shot and depended negligibly on $\mathcal{E}_L$ up to twice the energy used in experiments (see Supplementary Fig. 5, red data points). Pulse-to-pulse variations and energy dependence were stronger in the 4 ps, 0.5 TW configuration (see Supplementary Fig. 5, green data points), but remained at 4 ± 0.5 ps at energies used in the experiments.

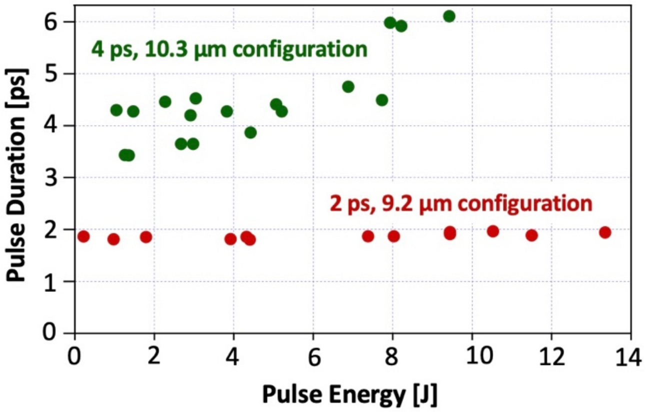

Supplementary Fig. 5. Duration of CO_2 laser pulses. Results of pulse duration (τ_L) measurements based on single-shot autocorrelation [2] for the 2 ps, 9.2 μm (red data points) and 4 ps, 10.3 μm (green data points) CO_2 laser configurations.

References for Supplementary Material

[1] Kumar, P. *et al*., Simulation study of CO_2 laser-plasma interactions and self-modulated wakefield acceleration. *Phys. Plasmas* **26**, 083106 (2019). https://doi.org/10.1063/1.5095780.

[2] Polyanskiy, M. H., Pogorelsky, I. V. , Babzien, M., and Palmer, M. A., Demonstration of a 2 ps, 5 TW peak power, long-wave infrared laser based on chirped-pulse amplification with mixed-isotope CO_2 amplifiers. *OSA Continuum* **3**, 459-472 (2020). https://doi.org/10.1364/)OSAC.381467.
